# Genetic Diversity of the Genus *Cosavirus* in the Family *Picornaviridae*: A New Species, Recombination, and 26 New Genotypes

**DOI:** 10.1371/journal.pone.0036685

**Published:** 2012-05-16

**Authors:** Beatrix Kapusinszky, Tung G. Phan, Amit Kapoor, Eric Delwart

**Affiliations:** 1 Blood Systems Research Institute, San Francisco, California, United States of America; 2 Department of Laboratory Medicine, University of California San Francisco, San Francisco, California, United States of America; 3 Department of Viral Diagnostics, National Center for Epidemiology, Budapest, Hungary; 4 Center for Infection and Immunity, Columbia University, New York, United States of America; Institute of Infectious Disease and Molecular Medicine, South Africa

## Abstract

The proposed viral genus human *Cosavirus* (HCoSV) consists of diverse picornaviruses found at high prevalence in the feces of children from developing countries. We sequenced four near-full length genomes and 45 partial VP1 region from HCoSV in human feces from healthy children and children with acute flaccid paralysis in Pakistan, Nigeria and Tunisia and from healthy and diarrhetic adults in Nepal. Genetic analyses of the near-full length genomes revealed presence of a new candidate cosavirus species provisionally labelled as species F (HCoSV-F). A HCoSV genome showed evidence of recombination between species D and E viruses at the P1/P2 junction indicating that these viruses may be reclassified as a single highly diverse species. Based on genetic distance criteria for assigning genotypes corresponding to neutralization serotypes in enteroviruses we identified 26 new HCoSV genotypes belonging to species A, D, and E. The detection of a large number of HCoSV genotypes based on still limited geographic sampling indicates that the phenotypic effects of cosaviruses on infected subjects are likely to be as highly diverse as those of human enteroviruses.

## Introduction

Cosavirus is a proposed new genus in the family *Picornaviridae* (www.picornaviridae.com) originally identified in 2008 in the feces of South Asian children with non-polio acute flaccid paralysis (AFP) [1]. HCoSV were found at high prevalence in feces of both healthy (44%) and paralyzed (49%) children from Pakistan [1]. Cosavirus was also detected in an Australian child with acute diarrhea [2] and in Chinese children with (3.2%) and without (1.6%) diarrhea [3]. Analysis of untreated sewage water also showed cosaviruses to be present in the United States [4]. Low viral loads of cosavirus were also recently reported in the feces of healthy Brazilian children from a community child-care center (49% in 2008 and 6.5% in 2011) and with gastroenteritis in a pediatrics department (3.6%) [5]. A single case of cosavirus infection was also reported in an adult with diarrhea in Thailand [6].

Cosaviruses, whose closest picornavirus relatives are cardioviruses and senecavirus have been tentatively classified into four distinct species labeled HCoSV-A to -D [1]. A fifth species (HCoSV-E) has also been reported in Australia [2].

The wide genetic diversity of this recently characterized viral genus complicates disease association studies since different viral species and serotypes may be expected (as is the case for other picornaviruses such as the extensively studied enteroviruses) [7] to have very different clinical impact on infected subjects. Here we extend our knowledge of the genetic diversity of human cosaviruses causing a highly common enteric infection of children in developing countries [1].

**Figure 1:**
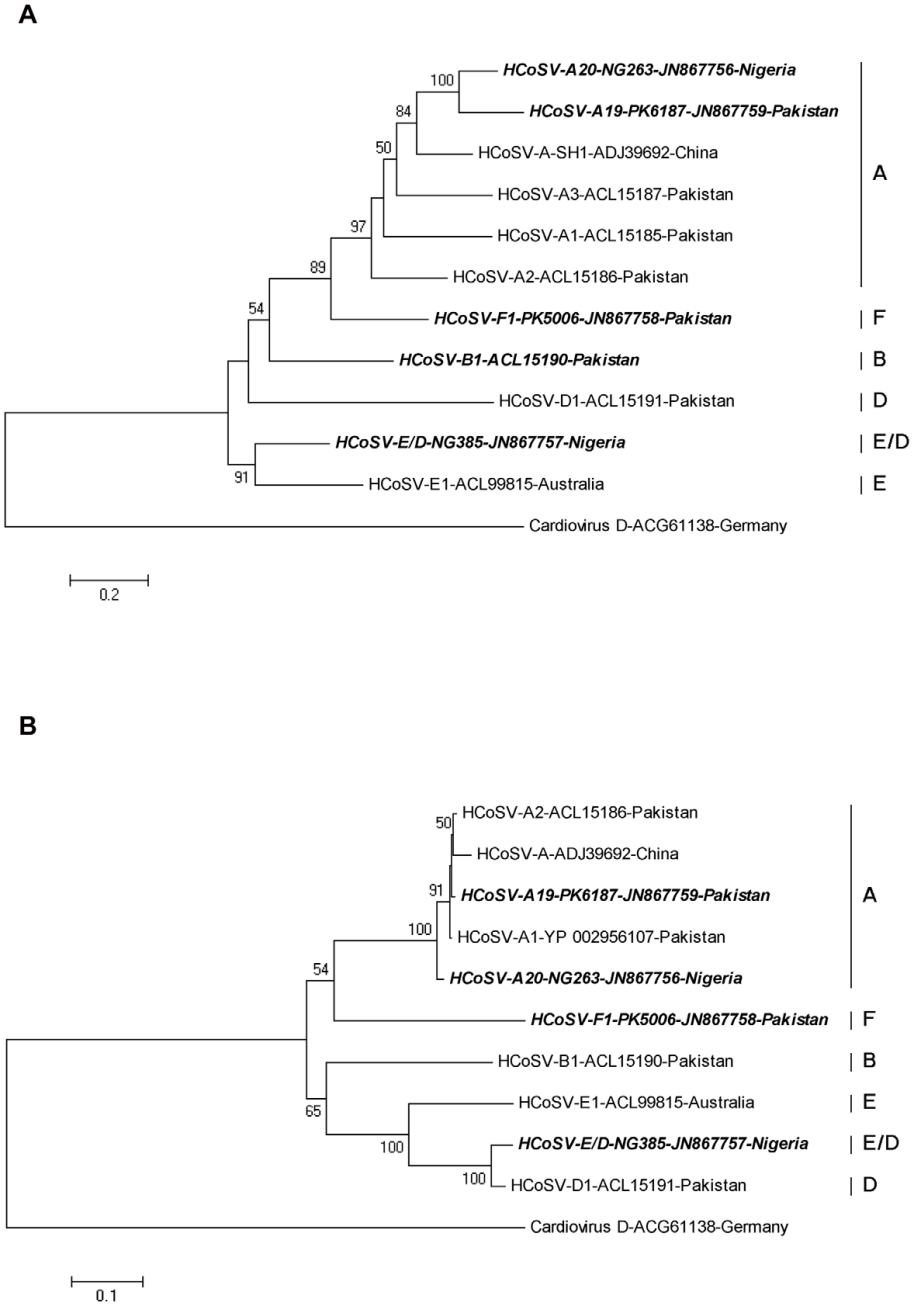
Phylogenetic analyses of P1 and 3D amino acid sequences of cosaviruses. Phylogenetic tree was constructed by the maximum-likelihood method. Species C was not included as no P1, and complete 3D regions are yet availabe. (A) Analysis of P1 region (B) Analysis of 3D region. Sequences from NG385 are labeled as species E/D due to its recombinant nature. Complete genomes from this study are indicated in bold. Cardiovirus D-ACG1138-Germany was used as an outgroup.

### Biological samples and methods

Stool samples used for cosavirus sequencing were collected by the WHO Collaborating Centers for Poliovirus and Enterovirus Surveillance in Pakistan (n = 2), Nigeria (n = 26), Tunisia (n = 5) and from the Armed Forces Research Institute of Medical Sciences, Enteric disease Department in Bangkok for Nepalese stool samples (n = 10). Feces samples from WHO collaborating group were from children under 15 years of age with acute flaccid paralysis or from healthy contacts of such patients. Nepalese samples were from adults with unexplained gastroenteritis and healthy adults.

**Figure 2:**
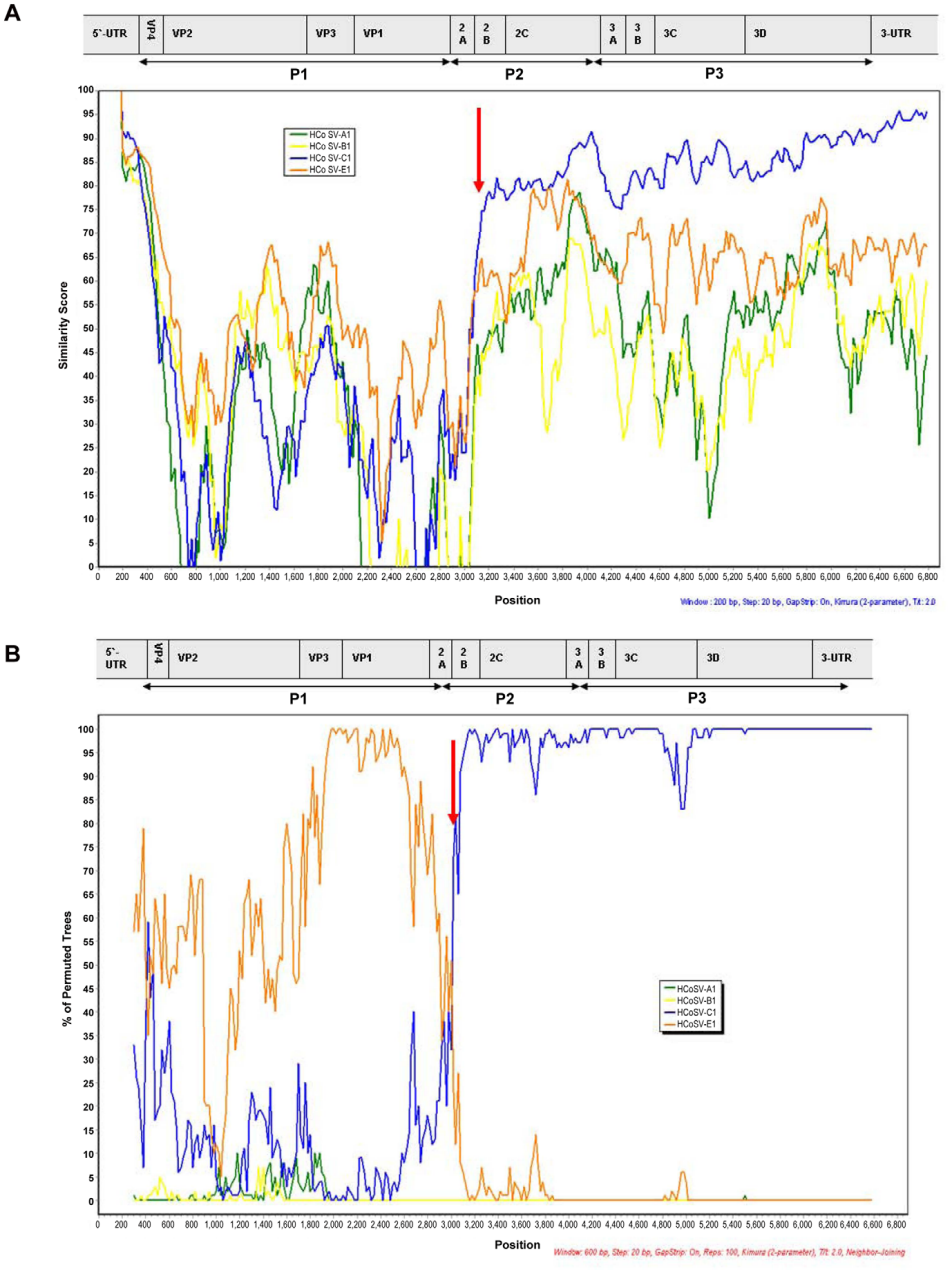
Recombination analyses. (A) Sliding window SimPlot graph generated by using NG385 sequence against all cosavirus species with complete genomes (HCoSV-A, -B, -D, -E). (B) Bootscan analysis of NG385 in comparison to other cosaviruses. Red arrow indicates the predicted recombination site in 2B of the non-structural region P2.

cDNA were generated with Superscript III (Invitrogen) and random primers. RT-PCR reactions were performed with LA*Taq* (Takara) to bridge pairs of separated cosavirus sequences previously derived by viral metagenomics [1], [8], [9]. The 5′ and 3′ RACE was used to extend sequences towards the viral genome extremities. PCR products were directly sequenced by primer walking.

Initial cosavirus detection was done using 5′UTR RT-nested PCR on nucleic acids extracted from feces as previously described [1]. The capsid sequences were generated by RT nested PCR using primers over the most conserved nucleotide regions. Upstream primers were positioned over VP3 and downstream primers were positioned over the 3′ end of VP1 as the immediate downstream region was too highly variable for consensus primer design. Primers for the first PCR round were VP1-INO-F1 (GAICARGCIATGATGGGIAC) and VP1-INO-R2 (GCIGGICCIGGRTTKGWYTC) in standard PCR conditions with first 15 cycles annealing at 60°C to 45°C (1°C decrease every cycle) followed by 25 cycles annealing at 54°C. Second PCR round used PCR primers VP1-INO-F1-2 (GCCATGATGGGIACITWYDCIATITGGGA) and VP1-INO-R3 (TARTCIGGRTAICCRTCRAA) in standard PCR conditions with first 10 cycles annealing at 60°C to 50°C (1°C decrease every cycle) followed by 30 cycles annealing at 50°C. I is for inosine, mixtures of bases are from the IUB nucleotide codes. A 904 bases amplicon was generated (from nucleotide position 2628 to 3531 on cosavirus reference genome HCoSV-A1, accession number NC_012800.1) and directly sequenced. Protein sequences aligned for phylogenetic analysis ranged in size from 272 to 281 amino acids spanning a portion of VP3 (VP3 amino acid 167 to 233 and VP1 (VP1 amino acid 1 to 206) in reference genome HCoSV-A1. The VP1 segment is 87 amino acids short of its carboxyl termini. We refer to these sequences as VP1*. Amplification products were extracted by QIAquick Gel Extraction kit (QIAGEN) and were directly sequenced by primer walking. Confirmation of the recombination site in NG385 was performed by direct RT-PCR and re-sequencing over the recombination point.

**Figure 3:**
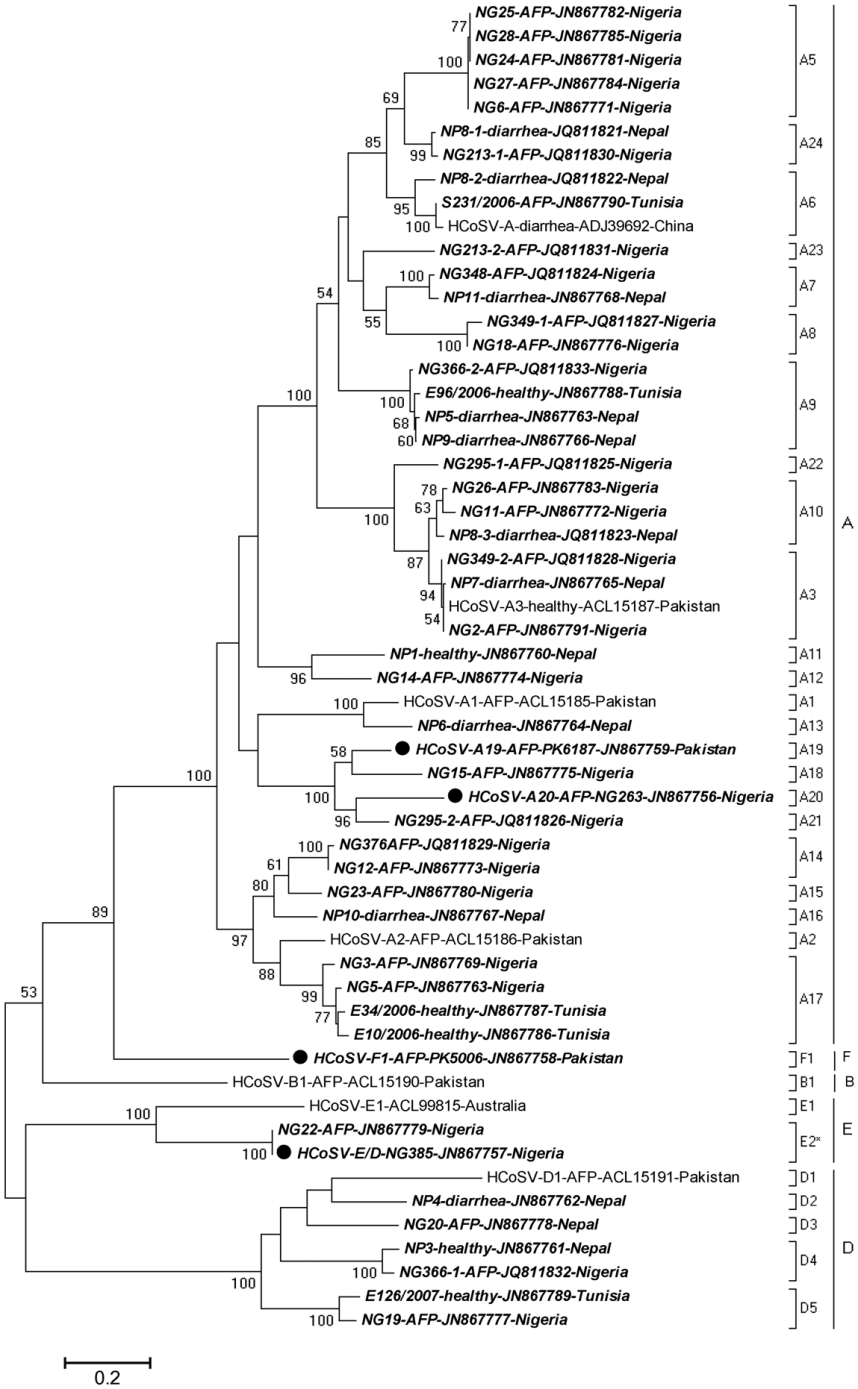
Phylogenetic anaylsis by Maximum Likelihood method. The percentage of trees in which the associated cosavirus VP1* clustered together is shown next to the branches with 50% cutoff. A discrete Gamma distribution was used to model evolutionary rate differences among sites. The tree is drawn to scale, with branch lengths measured in the number of substitutions per site. Cosaviruses sequences from this study with near full genomes are indicated by black dots and new VP1* sequences are in bold italics. The health status of individuals is included in the strain name. Pre-existing cosavirus sequences from Genbank are in regular font.

### Sequence analysis and recombination detection

DNA fragments were assembled into genome using Sequencher 5.0 program (Genecodes Corp.). Sequence alignments were constructed by MUSCLE [10] with a maximum of 64 iterations. Phylogenetic trees was generated using Maximum likelihood analysis, WAG substitution model and Gamma distribution estimated in Mega 5.0 [11]. Identity plot analyses were performed in BioEdit.

Similarity plots among the aligned nucleotide sequences were generated using SimPlot, version 3.5.1. [12]. The level of similarity in window of 200 bp was calculated by the Kimura two-parameter method. To detect potential recombination aligned sequences were subsequently analyzed by using the Bootscanning method; the neighbor-joining algorithm was run with 100 pseudoreplicates implemented in SimPlot.

These studies were approved by the UCSF Committee on Human Research. Informed consent was not required as samples were pre-existing and anonymized.

## Results

The ICTV states that enteroviruses sharing >70% amino acid identity in P1 and >70% amino acid identity in 2C and 3CD regions belong to the same species [13]. Based on this criteria four cosavirus species were initially proposed (HCoSV-A to D) that shared 48–55% amino acid identity in the P1 region and 63–72% identity in the 3D region [1]. A cosavirus genome from Australia, related to its closest relative HCoSV-D1 by 51% amino acid identity in the P1 region, 88% in 2C, and 77% in 3CD, was proposed as the fifth species HCoSV-E [2].

Starting with partial genome segments generated by random RT-PCR during prior metagenomic search for new viruses [1], [8], [9] we selected four samples with divergent HCoSV sequences for near-full genome sequencing by linking fragments with RT-PCR and using 5′ and 3′ RACE. A 6656 bases long section without polyA tail of a new HCoSV genome was obtained from a stool sample from a Pakistani child with non-polio acute flaccid paralysis including 182 bp of the 5′UTR, the entire predicted polyprotein of 2128 aa and 110 bp at the 3′ UTR (PK5006, GenBank: JN867758). Phylogenetically the P1 and 3D regions of this genome formed deep branches relative to the other cosavirus species (Figures 1A, B) and showed <70% identity in both P1 (≤60.6%) and 2C+3CD regions (≤68.8%) relative to other cosaviruses (Tables S1, S2). This genome was therefore sufficiently divergent to be proposed as the sixth cosavirus species HCoSV-F (Figures 1A, B).

### A cosavirus E/D recombinant

Another nearly complete cosavirus genome (6752 bases long) was amplified and sequenced from a Nigerian child with acute flaccid paralysis (NG385, GenBank: JN867757). The % P1 and 3D amino acid identies between this genome and those of other available cosavirus genomes indicated that it was closer to species E in P1 (63.9% versus ≤55.6% to other P1) but closer to species D in 2C+3CD region (95.2% versus ≤81% to other P1) (Tables S1, S2). The discordant relationship of NG385 to species D and E, depending on the genomic region, was confirmed by phylogenetic analysis of the P1 and 3D regions (Figures 1A, B). To test for the possibility of a recombination event the nucleotide sequences of cosavirus species A–E (species C being only partially sequenced was not included) and of NG385 were compared accross their genome using SimPlot and Bootscan analyses. The NG385 genome was closer to HCoSV-E in P1 until the start of the P2 regions downsream of which NG385 was more closely related to HCoSV-D. These results, typical of recombinantion, indicated that this genome is the first reported cosavirus recombinant (Figures 2A, B).

Picornavirus recombination is frequently observed within but not between viral species with the exception of exchange of the regulatory 5′UTR region [14]–[18]. The characterization of a recombinant genome between species HCoSV-E and HCoSV-D strains at the P1/P2 junction indicates that these two species may be re-classified as a single species. The % identity between the HCoSV-D1 and HCoSV-E prototypes (81% in 2C+3CD) was also higher than the 67.5–72% inter-species range observed between species A through D (Table S2). Therefore evidence of recombination between HCoSV-D and HCoSV-E and lower genetic distance between members of these proposed species than among other cosavirus species support their reclassification into the same cosavirus species.

Two other near-full length cosavirus genome were also sequenced: a 6635 base sequence of a Nigerian cosavirus (NG263, GenBank: JN867756) and 6950 bases from a Pakistani cosavirus (PK6187, GenBank: JN867759). Both of these genomes fell squarely within species A in both P1 and 2C+3CD regions.

### Cosavirus genotypes/serotypes

A numerous enterovirus serotypes have been genetically characterized based on VP1 sequence and variants showing greater than 88% amino acid identity in their VP1 have been shown to belong to the same antibody neutralization serotype [19]. Using this “≥88% criterion”, the previously sequenced viruses belonging to cosavirus species A could be sub-divided into 3 different VP1 genotypes assumed to correspond to serotypes (HCoSV-A1 through HCoSV-A3) [1]. Together with another species A strain from China [3] four VP1 of HCoSV-A species were avaible in GenBank. For HCoSV-B, -D and E only a single VP1 genotype has been described. The complete P1 region of HCoSV-C remains unsequenced and its species designation is based solely on 2C and 3CD analyses.

### 26 new genotypes

To improve our understanding of cosavirus diversity we screened fecal samples from 3 countries using a nested RT-PCR targeting the 5′UTR of cosaviruses. Feces from children under 15 years of age with non-polio acute flaccid paralysis from Nigeria showed a cosavirus prevalence of 40% (71/177 tested). A similar cohort from Tunisia showed a prevalence of 32% (61/192) while feces from healthy contacts of AFP patients showed a prevalence of 35% (67/192). Feces from adults with diarrhea and healthy adults in Nepal (described in [8]) showed a prevalence of 12% (12/100) and 15% (15/100) respectively. Nested RT-PCR of the capsid region (VP1*) was then performed on the 5′UTR positive samples using the PCR primers described in Materials and Methods generating 45 capsid VP1* (28% of VP3 and 72% of VP1) sequences, which together with the near full genomes provided 49 new VP1* sequences.

Phylogenetic analysis showed the dominance of HCoSV-A species (82% or 40/49 VP1*) followed by species D (12% or 6/49 VP1*). One VP1* sequence from Nigeria (NG22) was identical to the VP1* of the Nigerian NG365 the E/D recombinant. No VP1* related to that of the single strain of HCoSV-B was detected. In five cases co-infection with multiple cosaviruses were observed based on mixed electrophoretic peaks during Sanger sequencing of the PCR amplicons necessitating subcloning and sequencing of multiple plasmid inserts. Three cases of co-infection with two different genotypes of species A (NG213, NG295, NG349), one case of double infection with a species A and a species D genotypes, and one case of a triple infection with three species A genotypes (NP8) were detected. Using the genetic distance criteria of less than 88% amino acid identity in VP1 for defining a new enterovirus genotype/serotype [19], we identified a total of twenty new genotypes in cosavirus species A, four new genotypes in species D, one new genotype in species E (E2* the E/D recombinant), and one genotype in proposed species F (Figure 3). Therefore, counting all currently reported cosaviruses, there are a current total of 33 genotypes. A similar dominance of species A followed by species D was also reported in Brazil using partial 5′UTR sequence [5].

Some genotypes were found in a single country (eg. A5 from Nigeria) while others were found in multiple countries (eg. A3 from Pakistan, Nepal, and Nigeria). Some genotypes such as A5 were found exlcusively in AFP cases from Nigeria but the absence of control samples from healthy children precludes any definitive conclusion regarding A5 association with AFP. Cosavirus genotype A17 was found in two children with AFP from Nigeria, but also in two healthy children from Tunisia. Because of the extensive genetic diversity of cosaviruses in the three countries tested larger cases/control studies will be required to identify a link between any particular cosavirus genotype(s) and AFP or diarrhea.

### Conclusion

We report here on the genetic diversity of cosaviruses from four countries. Based on VP1* sequences, HCoSV-A appears to be the most common species followed by HCoSV-D a result similar to that seen with 5′UTR and 3D sequencing [1]. The high level of genetic diversity in the two most sampled cosavirus species (HCoSV-A currently with 24 genotypes and HCoSV-D currently with 5 genotypes) based on limited sampling indicates that their diversity may eventually reach that of human enteroviruses, which after decades of studies currently stands at 22 VP1 genotypes for HEV-A, 60 for HEV-B, 21 for HEV-C and 4 for HEV-D (www.picornaviridae.com). Based on genetic distance criteria and the detection of recombination in HCoSV-E/D-NG385 strain we also conclude that the previously reported species E could be reclassified as a genotype within species D with whom it shares a closely related P2 and P3 regions. A new cosavirus species, whose prototype is provisionally labeled HCoSV-F1-PK5006, was found in a Pakistani sample.

When no distinction was made between species or genotypes/serotypes no difference were found in overall cosavirus prevalence between cases of AFP and healthy children in Pakistan [1] or in Tunisia. A higher prevalence of cosaviruses was also not detected in the feces of adults with diarrhea in Nepal versus healthy controls, a result in keeping with the recent lack of association between gastroenteritis and generic cosavirus infections in Brazilian children [5].

The large number of cosavirus genotypes/serotypes reported here indicates that cosaviruses may exhibit a large range of phenotypic effect on their human hosts as do the similarly diverse enteroviruses [7]. Demonstrating pathogenicity or absence of pathogenicity for any cosavirus genotype will require VP1 sequencing in both a large number of diseases cases such as unexplained diarrhea, AFP or other conditions as well as in a large number of epidemiologically matched healthy controls in order to detect a measurable difference in genotype frequencies between groups.

## Supporting Information

Table S1 **Percent amino acid identity in P1 region of cosaviruses.**
(DOC)

Table S2 **Percent amino acid identity in 2C and 3CD regions of cosaviruses.**
(DOC)
