# Supplementary material for: Genetic Diversity of the Genus Cosavirus in the Family Picornaviridae: A New Species, Recombination, and 26 New Genotypes
Source: PLoS One. 2012 May 16;7(5):e36685. doi: 10.1371/journal.pone.0036685 (PMC3353919; doi:10.1371/journal.pone.0036685)
Supplement: Table S1 — Percent amino acid identity in P1 region of cosaviruses. (DOC) [file pone.0036685.s001.doc]

| **P1** | **HCoSV-A19 PK6187** | **HCoSV-A20 NG263** | **HCoSV-E/D NG385** | **HCoSV-F PK5006** | **HCoSV-A1** | **HCoSV-A2** | **HCoSV-A3** | **HCoSV-B1** | **HCoSV-D1** | **HCoSV-E1** |
| --- | --- | --- | --- | --- | --- | --- | --- | --- | --- | --- |
| **HCoSV-A1** | *64* | *63.2* | *52.9* | *57.9* | *ID* | *67.7* | *65.7* | *54.9* | *45.9* | *52.4* |
| **HCoSV-A2** | *62.4* | *65.4* | *53.8* | *60.6* | *67.7* | *ID* | *65.8* | *57.3* | *47.4* | *52.1* |
| **HCoSV-A3** | *61.7* | *64.2* | *52.4* | *57.1* | *65.7* | *65.8* | *ID* | *0.527* | *45.9* | *51.3* |
| **HCoSV-B1** | *51.5* | *53.7* | *55.6* | *55.5* | *54.9* | *57.3* | *52.7* | *ID* | *48.6* | *54.9* |
| **HCoSV-D1** | *43.6* | *46.5* | *48.5* | *47* | *45.9* | *47.4* | *45.9* | *48.6* | *ID* | *50.2* |
| **HCoSV-E1** | *49.2* | *50.7* | *63.9* | *49.7* | *52.4* | *52.1* | *51.3* | *54.9* | *50.2* | *ID* |

**Table S1.**
