# Supplementary material for: Genetic Diversity of the Genus Cosavirus in the Family Picornaviridae: A New Species, Recombination, and 26 New Genotypes
Source: PLoS One. 2012 May 16;7(5):e36685. doi: 10.1371/journal.pone.0036685 (PMC3353919; doi:10.1371/journal.pone.0036685)
Supplement: Table S2 — Percent amino acid identity in 2C and 3CD regions of cosaviruses. (DOC) [file pone.0036685.s002.doc]

**Table S2.**

| **2C+3CD** | **HCoSV-A19 PK6187** | **HCoSV-A20 NG263** | **HCoSV-E/D NG385** | **HCoSV-F PK5006** | **HCoSV-A1** | **HCoSV-A2** | **HCoSV-B1** | **HCoSV-D1** | **HCoSV-E1** |
| --- | --- | --- | --- | --- | --- | --- | --- | --- | --- |
| **HCoSV-A1** | *98.8* | *95.9* | *68.5* | *68.6* | ID | *98.8* | *72* | *68.5* | *68.7* |
| **HCoSV-A2** | *99.1* | *95.4* | *68.4* | *68.8* | 98.8 | ID | 71.8 | 68.4 | 68.8 |
| **HCoSV-B1** | *72.1* | *71.9* | *67.9* | *66.4* | 72 | 71.8 | ID | 68 | 67.5 |
| **HCoSV-D1** | *68.8* | *69.5* | *95.2* | *64.1* | 68.5 | 68.4 | 68 | ID | 81 |
| **HCoSV-E1** | *69.1* | *69.5* | *81* | *64.4* | 68.7 | 68.8 | 67.5 | 81 | ID |
